# Preparation of Ni/C porous fibers derived from jute fibers for high-performance microwave absorption†

**DOI:** 10.1039/d0ra06817a

**Published:** 2020-10-06

**Authors:** Wanxi Li, Fang Guo, Xiaoqin Wei, Yien Du, Yongqiang Chen

**Affiliations:** College of Chemistry and Chemical Engineering, Jinzhong University Jinzhong 030619 P. R. China liwanxi1986@163.com duyien124@163.com chenyongqiang82@126.com

## Abstract

Composites obtained by incorporating magnetic nanoparticles into porous carbon materials are promising in serving as microwave absorbing materials. In this study, Ni/C porous fibers were successfully synthesized through a simple *in situ* template method by using low-cost jute fibers as carbon source and template. The results showed that the Ni nanoparticles were uniformly loaded on the surface and hollow porous structure of the Ni/C porous fibers. Meanwhile, the content and size of the Ni nanoparticles on the Ni/C porous fibers can be controlled. Due to a suitable filling content, the synergistic effect of dielectric loss, interface polarization loss, magnetic loss and porous structure of the Ni/C porous fibers, an excellent microwave absorption performance was achieved. The minimum reflection loss value reached −43.0 dB, and a reflection loss value less than −10 dB was in the frequency range of 11.2–16.1 GHz with 2.0 mm thickness. In particular, under matching thickness (1.5–3.5 mm), the values of all the reflection loss peaks were below −20.0 dB. It is believed that this work can not only provide a new way to design excellent carbon-based microwave absorbing materials, but also offer an effective design strategy to synthesize biomass nanocomposites.

## Abstract 2

Ni/C porous fibers derived from jute fibers exhibited excellent microwave absorption performance.

## Graphical abstract

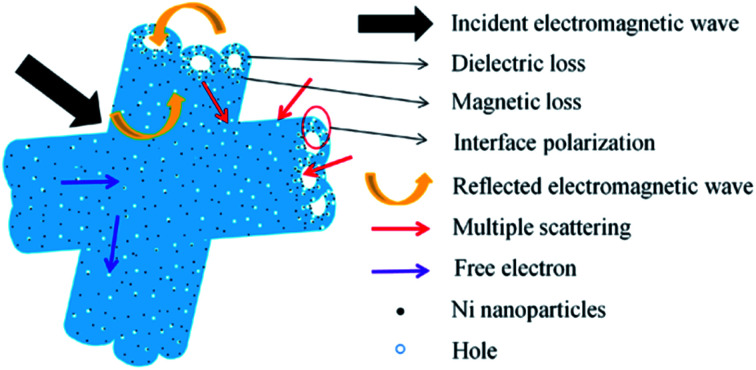

## 1. Introduction

With the widespread application of electronic and electrical equipment, wireless communication systems, and radar stealth technology, electromagnetic pollution and radiation problems have become increasingly serious, especially in the GHz band.^1,2^ The most effective way to solve these issues is to use high-efficiency microwave absorbing materials (MAMs) to absorb the electromagnetic waves and avoid strong electromagnetic wave reflection and secondary pollution.^3,4^ Hence, a considerable amount of effort has been devoted to the preparation of MAMs with multiple performance requirements.^5,6^

In recent years, carbon materials have been widely studied by researchers due to their good chemical and thermal stability, excellent dielectric properties, and low density. The optimized combination of magnetic nanoparticles and carbon materials can not only overcome the defects of high density of magnetic nanoparticles such as ferrite, magnetic metal, magnetic metal oxide, and alloy, but also improve the electromagnetic matching and synergistic effect, and achieve excellent microwave absorption performance.^7–9^ Much of the work has been reported recently in this field. For example, Chen *et al.* prepared NiFe_2_O_4_ hollow nanoparticles/graphene composite by a three-step synthesis method.^10^ Compared with NiFe_2_O_4_ nanoparticles and the mixture of NiFe_2_O_4_ nanoparticles and graphene, the NiFe_2_O_4_ hollow nanoparticles/graphene composite showed better microwave absorption performance. The minimum reflection loss (RL) was −40.9 dB and the RL value less than −10 dB was in the frequency range of 13.5–18 GHz. Ji *et al.* prepared FeCo/porous carbon fiber composite through electrospinning and subsequent heat treatment, and a minimum RL value of −56 dB was achieved.^11^ The excellent microwave absorption performance was mainly attributed to the strong dielectric loss and magnetic loss as well as the porous structure of the material. Although some progress has been made in the exploration of carbon-based composite MAMs, the carbon matrix materials used were mainly carbon fibers, carbon nanotubes, graphene and metal–organic framework compounds.^10–15^ The preparation method with cheap raw materials, simple process, and adjustable magnetic content in a wide range is still a challenging topic and difficulty in the research field of carbon-based MAMs.

Biomass is a kind of abundant renewable resource. In recent years, the efficient utilization of biomass has become a hot and difficult issue. After carbonizing biomass, porous carbon materials with multi-stage pore size distribution can be obtained.^16,17^ Recent studies showed that the porous structure was conducive to enhancing electromagnetic wave absorption, which was mainly attributed to the reflection and multiple scattering of electromagnetic wave in the porous media.^18,19^ The porous structure can not only reduce the density of the material, but also enhance the microwave absorption performance. Therefore, the research on biomass porous carbon materials would be a developing trend of new carbon-based MAMs.^3,20^ Natural cellulose is the one of the main biomass resources, which has good hygroscopicity and uniformity. Moreover, the cellulose contains a lot of –OH, which can provide many active sites for the loading of nanoparticles. Nowadays, the preparation of porous carbon materials using cellulose as precursor has aroused the attention of scholars in material fields, and the cellulose used to prepare carbon-based absorbents was mainly related to cotton.^21–23^ For example, Gong *et al.* prepared magnetic porous carbon using nickel nitrate and waste cotton as precursors.^21^ Due to the sufficient dielectric loss and a proper electromagnetic match, when the filler loading is only 10 wt% and the thickness of the absorber is only 1.9 mm, the minimum reflection loss could reach −40.5 dB at 15.8 GHz. Jute fiber, the second largest natural cellulose fiber, is low cost and ecological friendly. Jute fiber is long and thin with a diameter of 10–40 μm, and it has cell cavity. Therefore, jute fiber can be used as an economic and feasible raw material for preparing hollow porous carbon fiber and combining magnetic nanoparticles, which is expected to show excellent microwave absorption performance. However, there is no report in the current literature.

In this research, we report a simple *in situ* template method to prepare Ni/C porous fibers by using low-cost jute fiber as carbon source and template. By controlling the concentration of nickel nitrate, the precise and uniform loading of Ni nanoparticles on porous carbon materials can be realized, and the content and size can be further regulated in a wide range. As we expected, the Ni/C porous fibers showed excellent microwave absorption performance. This method has the advantages of low cost, abundant resources, simple technology, good repeatability, and suitable for large-scale preparation, which provides a new way to design excellent carbon-based MAMs.

## 2. Experimental

### 2.1 Materials preparation

In this work, Ni(NO_3_)_2_·6H_2_O was A.R. grade. The commercially available jute fiber was applied as raw material. Deionized water was used throughout the study. N_2_ (high purity, Taiyuan Taineng Gas Co., Ltd.) was used as a protective gas.

Ni/C porous fibers were synthesized by a simple *in situ* template method. In a typical procedure, 7.0 g of jute fiber was immersed in Ni(NO_3_)_2_ aqueous solution with the concentration of 0.2 mol L^−1^, 0.5 mol L^−1^ and 1.0 mol L^−1^, respectively. After soaking for 24 h, the wet jute fiber was removed with tweezers. When there was no solution dripped down, it was put into the evaporating dish and dried at 60 °C for 15 h. The product obtained above was then put in a ceramic boat and annealed in a horizontal tubular furnace at 700 °C, with a heating rate of 4 °C min^−1^ in N_2_ atmosphere. After holding for 2 h, the sample was naturally cooled to room temperature and removed. The samples obtained were Ni/C porous fibers, which were named as Ni/C-0.2, Ni/C-0.5, and Ni/C-1.0, respectively. As a contrast, the jute fiber was also annealed under the same conditions. The obtained sample was porous carbon fiber, which was named as PCF.

### 2.2 Materials characterization

Powder X-ray diffraction (XRD) patterns were obtained by a Shimadzu XRD-6100 diffractometer with Cu Kα radiation, employed a scan step of 0.02° in the 2*θ* range of 10° to 90°. Field-emission scanning electron microscopy (JSM-7900F, JEOL, Japan) with electron energy dispersion spectroscopy (EDS) and transmission electron microscope (JEOL, Japan) were used to characterize the morphology and structure of the samples. The thermogravimetric analysis was carried out on a STA6000 simultaneous thermal analyzer from room temperature to 700 °C at a heating rate of 10 °C min^−1^. The magnetic hysteresis loops were measured using a LakeShore 7404 vibrating sample magnetometer (VSM).

For microwave absorption measurement, the synthesized products were mixed with paraffin in the mass fraction of 33%, and then pressed into compact cylindrical specimens with an inner diameter of 3.00 mm and an outer diameter of 7.00 mm in the abrasive tool. The electromagnetic parameters (relative complex permittivity *ε*_r_ and relative complex permeability *μ*_r_) were measured by coaxial line method using an Agilent N5224A vector network analyzer (VNA) in the frequency range of 2–18 GHz. Based on the measured *ε*_r_ and *μ*_r_, the RL curves at different absorbent thickness were calculated according to the following equations:^24,25^

1 *Z*_in_ = *Z*_0_ (*μ*_r_/*ε*_r_)^1/2^ tanh[j(*2*π*fd*/*c*)(*μ*_r_*ε*_r_)^1/2^],

2 RL (dB) = 20 log |(*Z*_in_ − *Z*_0_)/(*Z*_in_ + *Z*_0_)|,

where *Z*_in_ is the input characteristic impedance of absorbent, *Z*_0_ is the characteristic impedance of free space, *f* is the frequency of electromagnetic wave, *d* is the thickness of absorbent, and *c* is the velocity of light.

## 3. Results and discussion

### 3.1 Phase and morphology analysis

**Fig. 1:**
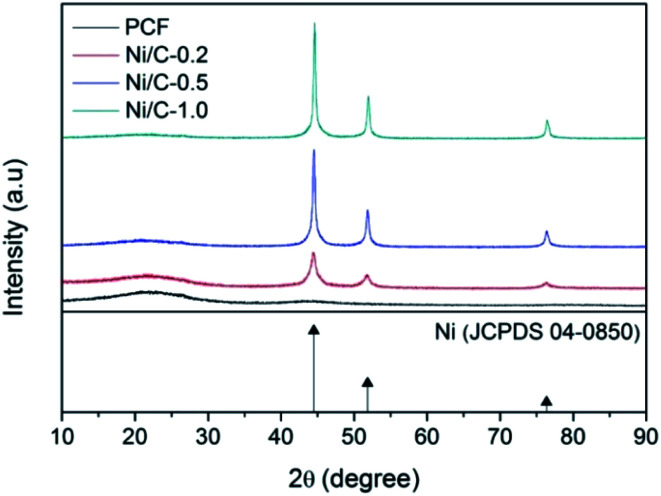
XRD patterns of PCF, Ni/C-0.2, Ni/C-0.5, and Ni/C-1.0.

Fig. 1 shows typical XRD patterns of the synthesized samples PCF, Ni/C-0.2, Ni/C-0.5, and Ni/C-1.0. For PCF, the broad peak at about 15°–30° indicates that the carbon component is amorphous.^11,26^ For Ni/C-0.2, Ni/C-0.5, and Ni/C-1.0, the diffraction peaks at 2*θ* = 44.51°, 51.85°, and 76.37° can be assigned to the (111), (200) and (220) crystalline planes of Ni (JCPDS No. 04-0850), respectively. That is to say, the Ni crystal is formed by *in situ* carbothermal reduction reaction. In addition, as can be seen from the XRD patterns, the Ni diffraction peaks enhance with the increase of Ni(NO_3_)_2_ concentration, indicating the increase of Ni nanoparticles content.

**Fig. 2:**
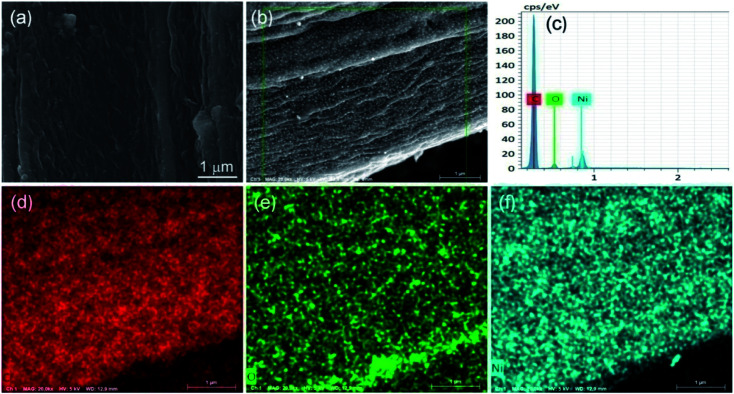
SEM image of PCF (a), SEM image of Ni/C-0.5 (b), EDX spectra of Ni/C-0.5 (c), and elemental mapping images (d–f) of Ni/C-0.5.

Fig. 2a displays the SEM image of PCF, and it presents smooth surface. Fig. 2b presents the SEM image of Ni/C-0.5, and it can be seen that many nanoparticles uniformly distribute on the surface of carbon fiber. Fig. 2c–f shows the EDX elemental maps of Ni/C-0.5. Clearly, the Ni element is uniformly dispersed on the Ni/C-0.5, which also indicates that the Ni nanoparticles generated are dispersed uniformly on the Ni/C fibers.

**Fig. 3:**
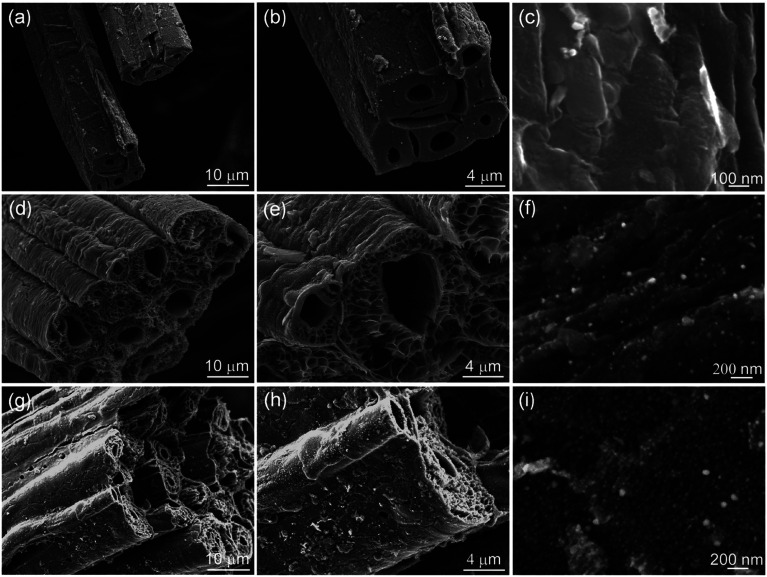
SEM images of (a–c) Ni/C-0.2, (d–f) Ni/C-0.5, and (g–i) Ni/C-1.0 in different magnification.

**Fig. 4:**
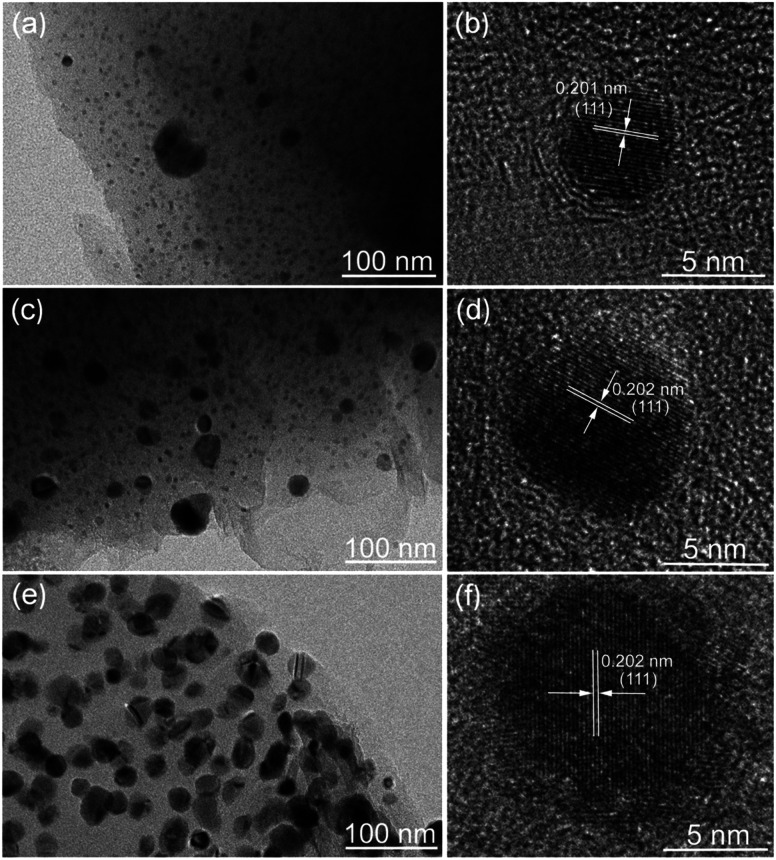
TEM and corresponding HRTEM images of (a and b) Ni/C-0.2, (c and d) Ni/C-0.5, and (e and f) Ni/C-1.0.

The morphology and structure of the Ni/C fibers were further characterized by SEM and TEM. Fig. 3 displays the representative SEM images of Ni/C-0.2, Ni/C-0.5, and Ni/C-1.0 in different magnification. It is clear that the Ni/C porous fibers have hollow internal cavities and porous structures, and the Ni nanoparticles are relatively dispersed on the surface and porous structure of the Ni/C porous fibers. Fig. 4 shows the TEM and corresponding HRTEM images of Ni/C-0.2, Ni/C-0.5, and Ni/C-1.0, from which the Ni nanoparticles on the Ni/C porous fibers have good dispersibility. The detailed particle size distribution of Ni nanoparticles on Ni/C-0.2, Ni/C-0.5, and Ni/C-1.0 was shown in Fig. S1.† For Ni/C-0.2, the Ni nanoparticles are highly dispersive, and the size of the vast majority of Ni nanoparticles is in the range of 4–8.5 nm (as shown in Fig. 4a and S1a†). Fig. 4b shows the corresponding HRTEM image of Ni nanoparticles in Ni/C-0.2, and the interplane distance of the lattice fringe is 0.201 nm, which can be identified as the (111) plane of Ni. For Ni/C-0.5, TEM image in Fig. 4c reveals that the size of the Ni nanoparticles is mainly 4–8.2 nm, and some larger nanoparticles with the size of 10–30 nm are formed. For Ni/C-1.0, as depicted in Fig. 4e and f, it is apparent that the size of the vast majority of Ni nanoparticles is in the range of 10–27.6 nm. On the basis of the above results, the possible synthesis mechanism of the Ni/C porous fibers is discussed. Firstly, the jute fibers have good hygroscopicity. When jute fibers are immersed in Ni(NO_3_)_2_ aqueous solution, Ni(NO_3_)_2_ aqueous solution is easy to permeate into the surface and pores of jute fibers. Then, the jute fibers are converted into porous carbon fibers under constant flow of N_2_ at high temperature, and the decomposition products of Ni(NO_3_)_2_ react with porous carbon fibers, and the Ni nanoparticles are deposited on porous carbon fibers. It is believed that the amorphous carbon and the porous structure prevent the agglomeration of the Ni nanoparticles. In addition, by changing Ni(NO_3_)_2_·6H_2_O to Co(NO_3_)_2_·6H_2_O in the synthesis route, Co nanoparticles can also be loaded on porous carbon fibers (see ESI Fig. S2 and S3†). This indicates that the *in situ* template method can also be used to load other nanoparticles on porous carbon fibers.

**Fig. 5:**
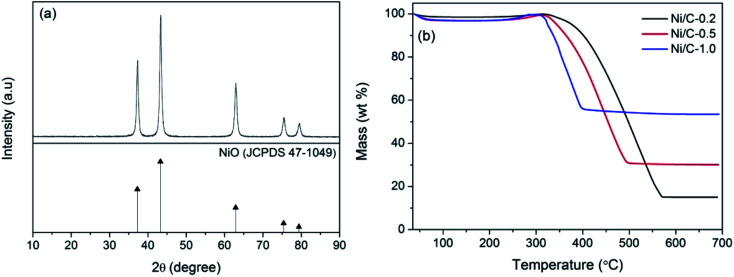
(a) XRD pattern of Ni/C-0.5 calcined at 500 °C for 2 h in air atmosphere, (b) TG curves of Ni/C-0.2, Ni/C-0.5, and Ni/C-1.0 in air atmosphere.

Fig. 5a shows the XRD pattern of Ni/C-0.5 calcined at 500 °C for 2 h in air atmosphere. There exist five main diffraction peaks at about 37.25°, 43.28°, 62.88°, 75.41°, and 79.41°, which are indexed to the (111), (200), (220), (311) and (222) crystalline planes of NiO (JCPDS No. 47-1049), respectively. Therefore, the thermogravimetric analysis method in air atmosphere can be used to estimate the contents of Ni nanoparticles on Ni/C-0.2, Ni/C-0.5, and Ni/C-1.0. From the TG curves in Fig. 5b, it can be seen that there is distinct weight loss, which is dependent on the combustion of carbon and the transform of Ni to NiO. When the carbon is completely burned in air and removed, the residual product will be only NiO. The concrete calculating methods are as follows: the atomic weight of Ni is 58.69; the atomic weight of NiO is 74.69; the percentage of residual mass is *M*; the weight percentage of Ni element in Ni/C porous fibers can be calculated by the following formula 58.69*M*/74.69. According to the TG curves and formula, the contents of Ni nanoparticles on Ni/C-0.2, Ni/C-0.5, and Ni/C-1.0 were 11.8 wt%, 23.5 wt%, and 41.8 wt%, respectively.

**Fig. 6:**
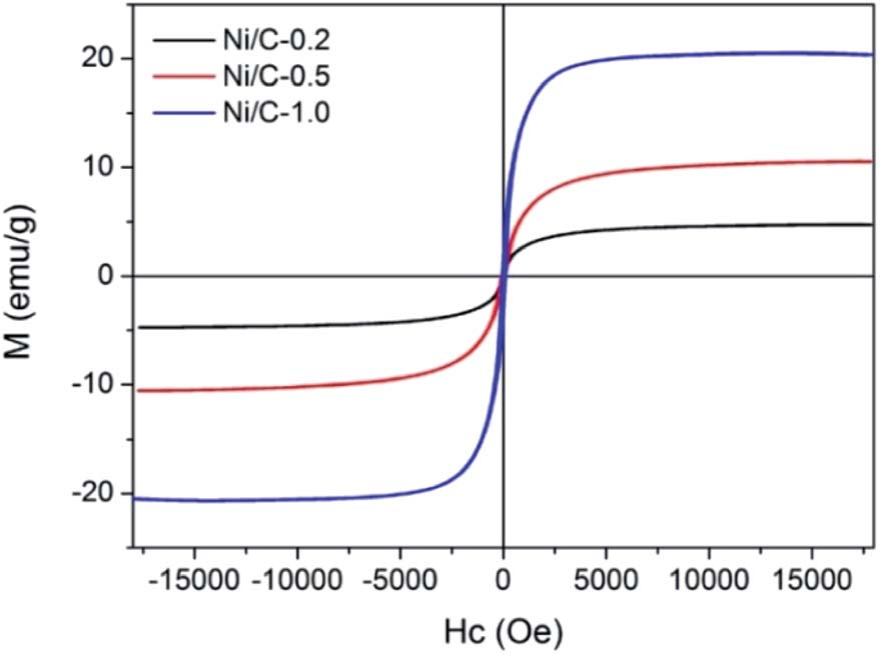
The hysteresis loops of Ni/C-0.2, Ni/C-0.5, and Ni/C-1.0 at room temperature.

The magnetic hysteresis loops of Ni/C-0.2, Ni/C-0.5, and Ni/C-1.0 were measured by VSM at room temperature. As indicated by the magnetic hysteresis loops in Fig. 6, all the three samples show typical ferromagnetic behavior. The saturation magnetization (*M*_S_) of Ni/C-0.2, Ni/C-0.5, and Ni/C-1.0 was 4.71, 10.53, and 20.33 emu g^−1^, respectively, which is highly dependent on the content of Ni nanoparticles on Ni/C porous fibers. In addition, the *M*_S_ value of Ni/C porous fibers is lower than that of pure Ni of 55 emu g^−1^,^27^ which is mainly attributed to the nonmagnetic porous carbon fibers and particle size of Ni nanoparticles.

### 3.2 Microwave absorption analysis

**Fig. 7:**
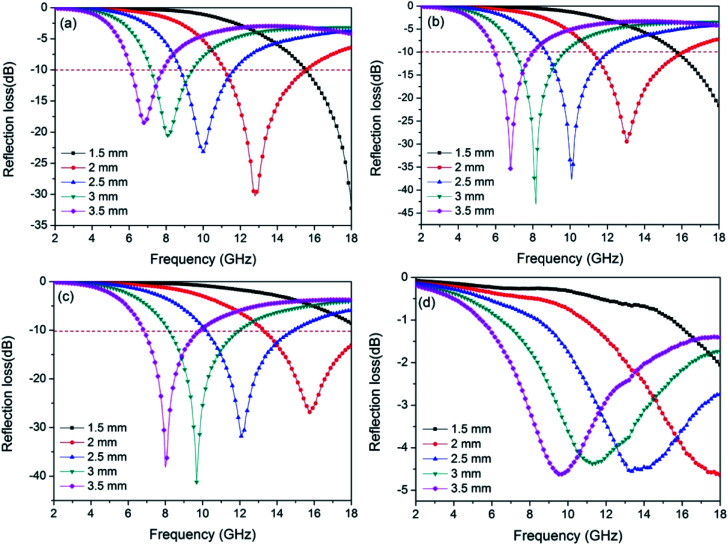
Frequency dependence of the reflection loss curves for (a) PCF, (b) Ni/C-0.2, (c) Ni/C-0.5, and (d) Ni/C-1.0 with different thicknesses.

Through the above results, using low-cost jute fiber as carbon source and template, Ni nanoparticles with different contents were successfully deposited on porous carbon fibers by an *in situ* template method. For investigating the microwave absorption performance of PCF and Ni/C porous fibers, the frequency dependence of the electromagnetic parameters were obtained from the VNA. It is generally true that the RL value of −10 dB represents 90% incident microwave is absorbed, and the effective bandwidth means the width of the frequency range when the RL is −10 dB. Fig. 7 displays the calculated RL curves of PCF, Ni/C-0.2, Ni/C-0.5, and Ni/C-1.0. It is generally believed that the minimum RL, the frequency position of the microwave absorption peak and the effective bandwidth are closely related to the thickness of the absorbent. Therefore, we calculated the RL curves for each sample with different thicknesses (1.5, 2.0, 2.5, 3.0 and 3.5 mm). As exhibited in Fig. 7, with the increase of the thickness of the absorbent, the RL peak gradually moves to the low frequency, which can be explained by the “geometrical effect”, meaning that the frequency position of the minimum RL is inversely proportional to the matching thickness.^28^ For PCF, the minimum RL is −30.3 dB with a thickness of 2.0 mm, and the effective bandwidth is 4.4 GHz (from 11.16 to 15.56 GHz), as shown in Fig. 7a. When the thickness is increased, the microwave absorption performance becomes poor. By contrast, the microwave absorption performances of Ni/C-0.2 and Ni/C-0.5 are improved, and the RL curves have a similar change trend (see Fig. 7b and c). For Ni/C-0.2, when the thickness is 2.0 mm, the effective bandwidth can reach up to 4.9 GHz (from 11.20 to 16.10 GHz), and the minimum RL is −43.0 dB. Especially, under the matching thickness (1.5–3.5 mm), a good microwave absorption performance (RL ≤ −10.0 dB) from 6 GHz to 18 GHz is obtained, and all of the values of RL peaks are below −20.0 dB. For Ni/C-0.5, the effective bandwidth achieves 4.72 GHz (from 13.28 to 18 GHz) with a thickness of 2.0 mm. When increasing the matching thickness to 2.5 mm, the effective bandwidth achieves 4.75 GHz (from 10.15 to 14.9 GHz). It is obvious that the Ni/C-0.5 shows broad effective bandwidth with the thicknesses ranging from 2.0 to 2.5 mm, and the application diversification can be realized in a wide thickness range. Fig. 7d shows the RL curve of Ni/C-1.0, and the effective bandwidth is 0, which means that the Ni content is not the more the better. As important reference parameters, the minimum RL and the effective bandwidth are compared with other reported carbon-based MAMs, as shown in Table 1. It is clear that the Ni/C porous fibers have broader effective bandwidth and stronger RL.^29–34^ Moreover, in contrast with the traditional microwave absorbents, the Ni/C porous fibers present the advantage of low density due to the existence of porous carbon fibers. Considering that our synthetic method is simple, low cost, and suitable for large-scale preparation, it is attractive for microwave absorption application.

**Table 1:** Microwave absorption performances of some reported carbon-based absorbents

Sample	Thickness (mm)	Effective bandwidth (GHz)	Minimum RL (dB)	References
CoFe_2_O_4_/graphene	2.0	3.7	−24.7	29
Fe_3_O_4_@C composites	2.0	4.9	−18.1	30
MWCNTs/Fe_3_O_4_	2.0	2.5	−29.8	31
Fe_3_C/C nanocapsules	2.0	4.0	−38.0	32
Ni/C nanocapsules	3.5	4.0	−34.0	33
Mesoporous Fe_3_O_4_/C	2.0	2.0	−18.1	34
Ni/C porous fibers	2.0	4.9	−43.0	This work

**Fig. 8:**
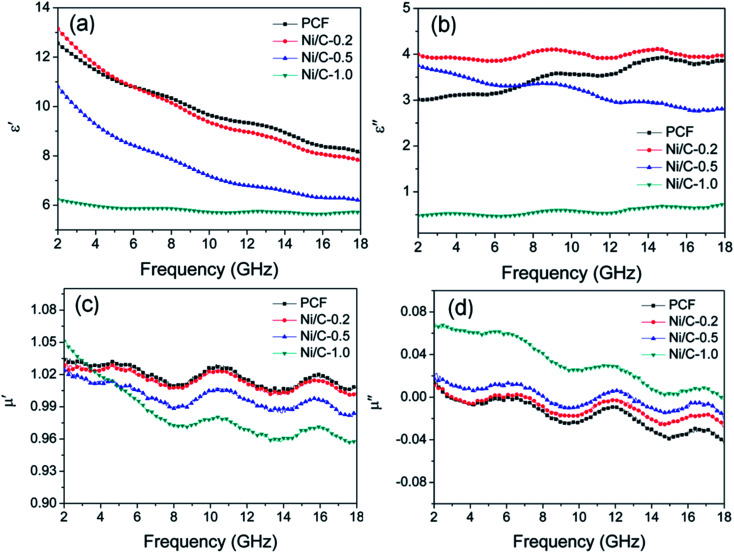
Frequency dependence of (a) the real part (*ε*′) and (b) imaginary part (*ε*′′) of complex permittivity, (c) real part (*μ*′) and (d) imaginary part (*μ*′′) of complex permeability.

**Fig. 9:**
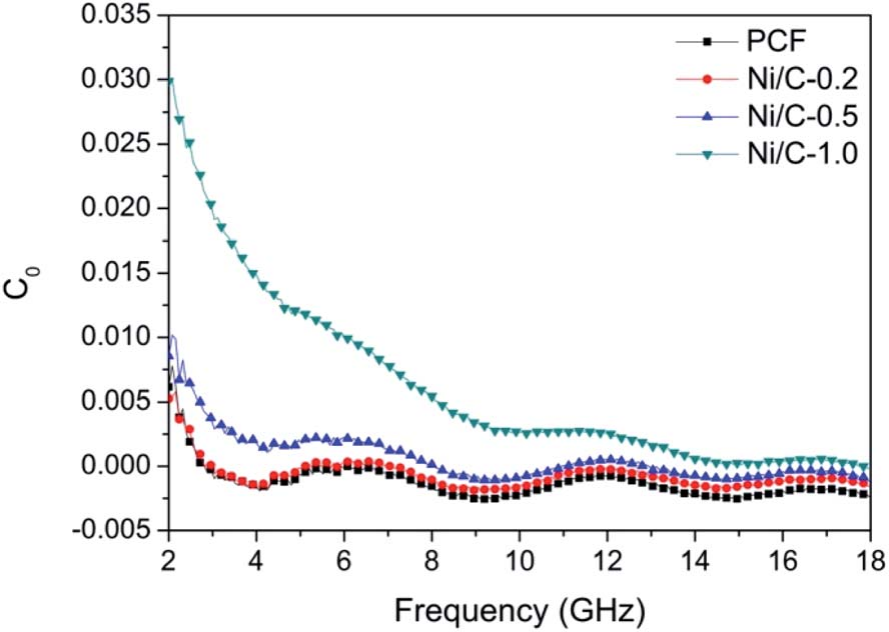
Values of *C*_0_ (*C*_0_ = *μ*′′*μ*′^−2^*f*^−1^) of PCF, Ni/C-0.2, Ni/C-0.5, and Ni/C-1.0.

According to the electromagnetic energy conversion principle, the microwave absorption of the absorbent is mainly determined by impedance matching and electromagnetic wave attenuation. The impedance matching is determined by the relative complex permittivity (*ε*_r_ = *ε*′ − j*ε*′′) and the relative complex permeability (*μ*_r_ = *μ*′ − j*μ*′′), and ensure that the electromagnetic wave can enter into the absorbent effectively. The real parts of the complex permittivity (*ε*′) and the complex permeability (*μ*′) stand for the stored electrical and magnetic energy, while the imaginary parts of the complex permittivity (*ε*′′) and the complex permeability (*μ*′′) correspond to the dissipation of electrical and magnetic energy.^35,36^ The electromagnetic wave attenuation is mainly reflected by the dielectric loss factor (tan *δ*_e_ = *ε*′′/*ε*′) and magnetic loss factor (tan *δ*_m_ = *μ*′′/*μ*′), and ensure that the absorbent can effectively attenuate the electromagnetic wave energy in its interior. Fig. 8 shows the frequency dependence of electromagnetic parameters of PCF, Ni/C-0.2, Ni/C-0.5, and Ni/C-1.0. As shown in Fig. 8a, the *ε*′ values of PCF, Ni/C-0.2, and Ni/C-0.5 tend to decrease continuously with the increase of frequency. However, the *ε*′ value of Ni/C-1.0 decreases slowly in the frequency range of 2–10 GHz and then remains stable with the increase of frequency. Moreover, the *ε*′ values of Ni/C-1.0 is the smallest, showing minimum electrical energy storage. The *ε*′′ values of PCF, Ni/C-0.2, Ni/C-0.5, and Ni/C-1.0 are shown in Fig. 8b, and all of the *ε*′′ values show two distinct vibration peaks in the frequency range of 6–12 GHz and 12–16 GHz. The *ε*′′ value of Ni/C-1.0 is smaller than that of PCF, Ni/C-0.2, and Ni/C-0.5, showing minimum electrical energy loss. Moreover, from the free electron theory, *ε*′′ = 1/(2π*ρfε*_0_), where *ρ* is the resistivity, *f* is the microwave frequency, and *ε*_0_ is the permittivity of vacuum.^37^ It can be speculated that a higher value of *ε*′′ corresponds to a lower resistivity. In this case, the loading of Ni nanoparticles on Ni/C porous fibers can enhance the conductivity, resulting in the increase of *ε*′′. However, if the loading content of Ni nanoparticles is too high, which may interrupt the conductive interconnection and reduce the conductivity of Ni/C porous fibers. For PCF, Ni/C-0.2, Ni/C-0.5, and Ni/C-1.0, the *μ*′ and *μ*′′ of complex permeability are displayed in Fig. 8c and d. It is clear that both *μ*′ and *μ*′′ show same trend with three obvious fluctuations. With increasing the content of Ni nanoparticles, the *μ*′′ values present an increasing trend, which is consistent with the content of Ni nanoparticles on Ni/C porous fibers and the VSM results. The low permeability is mainly due to the low loading of magnetic components and the low filling content of Ni/C porous fibers in paraffin matrix. Generally speaking, natural resonance, exchange resonance and eddy current effect are the main causes of magnetic loss in GHz band. Eddy current loss is related to the particle diameter (*d*), electric conductivity (*σ*) and vacuum permeability (*μ*_0_), which can be expressed by *μ*′′ ≈ 2π*μ*_0_*μ*′^2^*σd*^2^*f*/3. If the eddy current effect dominates the magnetic loss mechanism, the value of *C*_0_ = *μ*′′*μ*′^−2^*f*^−1^ remains constant with changing frequency.^38^Fig. 9 shows the *C*_0_–*f* curves of PCF, Ni/C-0.2, Ni/C-0.5, and Ni/C-1.0. It is obvious that they all show three obvious fluctuations in the frequency range of 4–8 GHz, 9–14 GHz and 15–18 GHz, which proves that the eddy current loss is not the main contributor to the magnetic loss. Besides, it can be concluded that the resonance peaks at 12 GHz and 16.5 GHz are related to the exchange resonance, and the peak at 6 GHz is attributed to the natural resonance.^39^ In addition, *μ*′′ has negative value in high frequency band. According to the Maxwell equations, the motion of free charges can produce AC electric field, and the magnetic field can be induced by AC electric field.^40^ Therefore, we speculate that the negative *μ*′′ value indicates that the magnetic energy is radiated out from the Ni/C porous fibers, and this negative *μ*′′ phenomenon has also been observed in many high conductivity absorbents.^41,42^

**Fig. 10:**
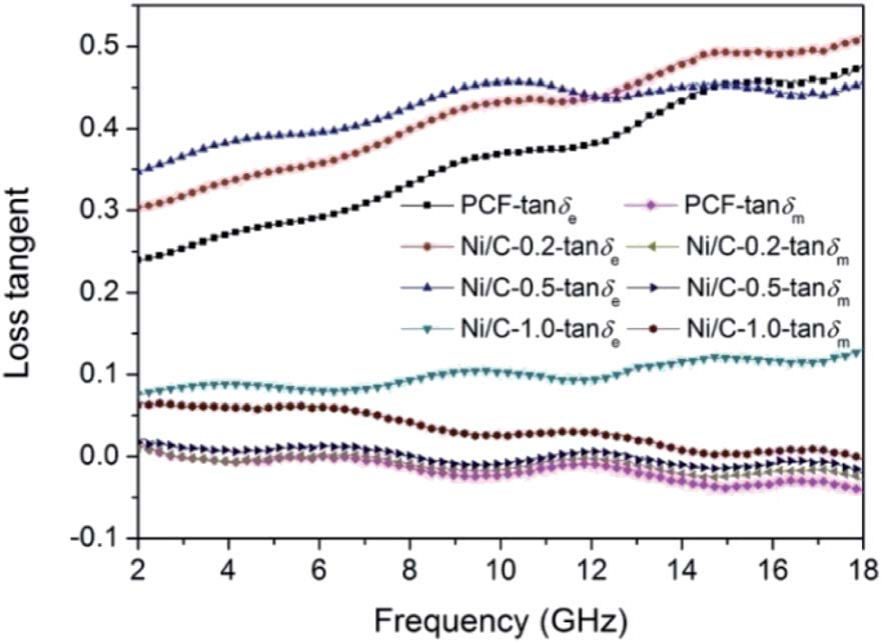
Frequency dependence of the loss tangent for PCF, Ni/C-0.2, Ni/C-0.5, and Ni/C-1.0.

Fig. 10 shows the loss tangent of PCF, Ni/C-0.2, Ni/C-0.5, and Ni/C-1.0. It is perfectly clear that the dielectric loss tangent shows an increasing trend with the increase of frequency. In the frequency range of 2–18 GHz, the tan *δ*_e_ values of the four samples are significantly greater than the tan *δ*_m_ values, indicating that the dielectric loss plays a major role in the electromagnetic loss, which is similar to other carbon-based MAMs.^43–45^ The tan *δ*_m_ follows the order of Ni/C-1.0 > Ni/C-0.5 > Ni/C-0.2 > PCF, which coincides with the VSM results. The tan *δ*_e_ and tan *δ*_m_ values of Ni/C-0.2 and Ni/C-0.5 are significantly higher than that of PCF, showing stronger electromagnetic loss. For Ni/C-1.0, although the tan *δ*_m_ is the higher than that of PCF, Ni/C-0.2, and Ni/C-0.5, the tan *δ*_e_ is too small, showing minimum electric energy loss and leading to weak microwave absorption. Consequently, introduction of appropriate content of Ni nanoparticles on Ni/C porous fibers can not only increase magnetic loss, but also strengthen dielectric loss, which can improve impedance matching and enhance microwave absorption performance. It is worth noting that the two curves of tan *δ*_e_ and tan *δ*_m_ have an opposite trend in the frequency range of 2–18 GHz, and this result can be explained by the coupling and modulation between dielectric behavior and magnetic behavior.^12,46,47^

**Fig. 11:**
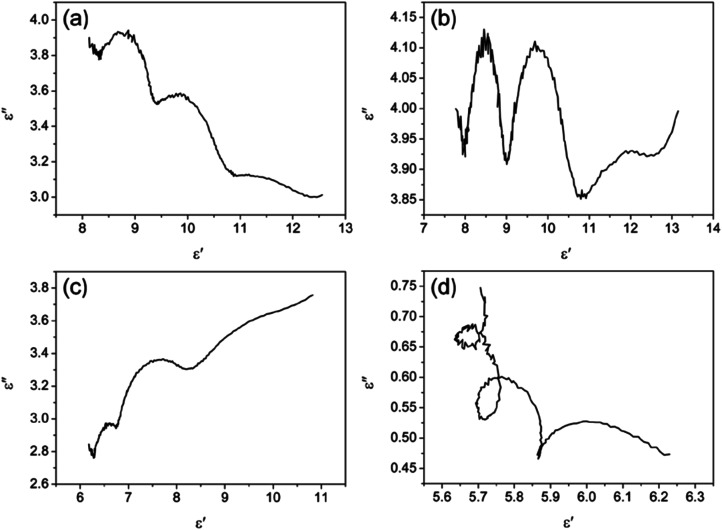
The curves of *ε*′ *versus ε*′′ for (a) PCF, (b) Ni/C-0.2, (c) Ni/C-0.5, and (d) Ni/C-1.0.

According to Debye theory, the dielectric loss mainly originates from conduction loss and polarization relaxation loss, and the polarization relaxation loss is responsible for the several fluctuations of curves of *ε*′′ and tan *δ*_e_.^48,49^ Seen from the above-mentioned SEM and TEM images in Fig. 3 and 4, the *in situ* template method can not only improve the dispersion of Ni nanoparticles on the Ni/C porous fibers, but also lead to a large number of interfaces among single porous carbon fibers and between Ni nanoparticles and porous carbon fibers. These multi-interfaces can cause interfacial polarization, and contribute to the polarization relaxation loss of the Ni/C porous carbon fibers. The interfacial polarization can be analyzed by Cole–Cole semicircle. According to Debye relaxation theory, the relationship of *ε*′ and *ε*′′ follows the following formula:^49–51^3
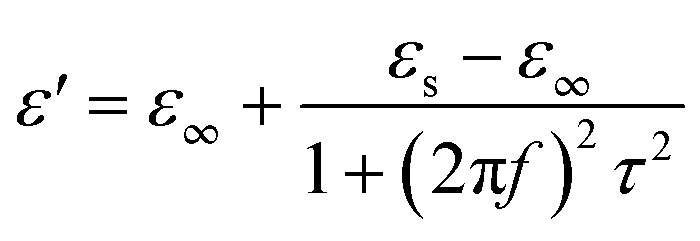
4
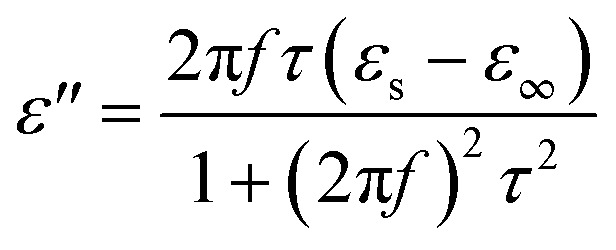
5
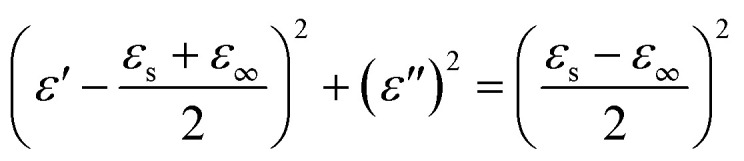
where *ε*_s_ represents the static permittivity, *ε*_∞_ stands for the relative dielectric permittivity at the high-frequency limit, *f* refers to the frequency, and *τ* indicates the polarization relaxation time. For the graph of *ε*′ *versus ε*′′, one Debye relaxation process corresponds to one semicircle (namely Cole–Cole semicircle), and the Cole–Cole semicircle is mostly due to the polarization relaxation loss. Fig. 11 shows the curves of *ε*′ *versus ε*′′ for PCF, Ni/C-0.2, Ni/C-0.5, and Ni/C-1.0 in the frequency range of 2–18 GHz, and all the four samples have more than one semicircle, showing evident polarization relaxation loss, which is good for dielectric loss. The *ε*′–*ε*′′ curves of PCF and Ni/C-0.5 have two Cole–Cole semicircles, while Ni/C-0.2 and Ni/C-1.0 have three and four Cole–Cole semicircle respectively, which indicates that the interface polarization effect of Ni/C-0.2 and Ni/C-1.0 is more obvious.

The filling content of Ni/C porous fibers in the paraffin matrix is also an important parameter affecting the microwave absorption performance. Fig. S4† shows the complex permittivity, complex permeability, loss tangent, and reflection loss curve for the specimen with 25 wt% Ni/C-0.2 in paraffin. It is clear that the dielectric loss tangent and magnetic loss tangent are lower than the specimen with 33 wt% Ni/C-0.2 in paraffin. The effective bandwidth is 4.3 GHz (from 13.7 to 18 GHz) with a matching thickness of 2 mm, and the minimum RL is −17.19 dB, which is poor than the specimen with 33 wt% Ni/C-0.2 in paraffin. Fig. S5† shows the complex permittivity, complex permeability, loss tangent, and reflection loss curve for the specimen with 50 wt% Ni/C-0.2 in paraffin. It can be noted that the complex permittivity is much higher than the specimen with 33 wt% Ni/C-0.2 in paraffin, and the effective bandwidth is 0 GHz. The poor microwave absorption performance is mainly attributed to the high complex permittivity and strong electromagnetic wave reflection. Therefore, the proper filling rate is 33 wt% with excellent microwave absorption (see ESI Fig. S4 and S5†).

**Fig. 12:**
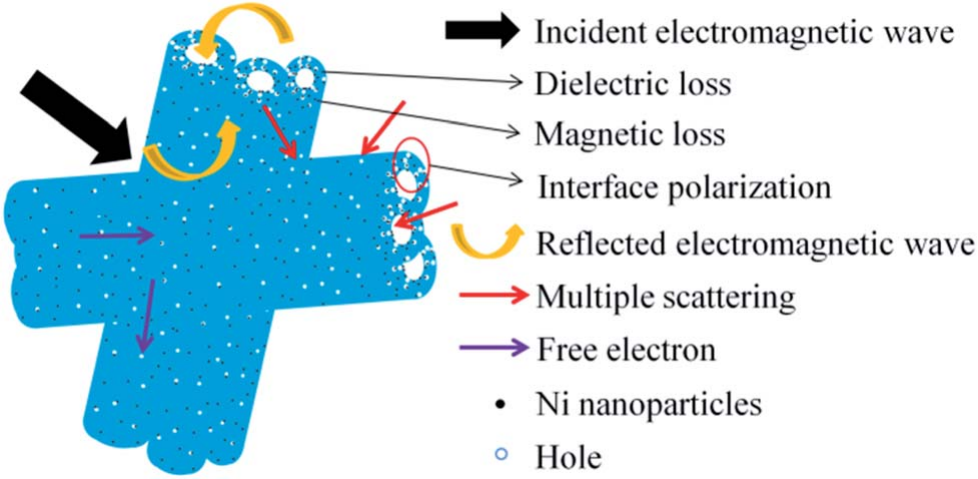
Schematic diagram of microwave absorption mechanism of the Ni/C porous fibers.

Based on the above experimental results and analyses, a possible microwave absorption mechanism of Ni/C porous fiber is elucidated in Fig. 12. Firstly, the proper filling content with moderate complex permittivity is beneficial to enhance the microwave absorption performance. And the introduction of Ni nanoparticles can not only improve the magnetic property of Ni/C porous fibers, but also improve the synergistic effect of Ni nanoparticles and porous carbon fibers. Secondly, porous carbon nanofibers can form conductive networks, which play an important role in dielectric loss, and the strong electromagnetic loss of Ni/C-0.2, Ni/C-0.5 can effectively enhance the microwave absorption performance. And the uniform dispersion of Ni nanoparticles on Ni/C porous fibers provides rich interfaces, which can greatly promote the interfacial polarization, resulting in the increase of dielectric loss. Finally, in the alternating microwave field, porous carbon nanofibers can provide a direct pathway for free electron transfer, and the transmitted microwave will be reflected and scattered in the porous media, which eventually can be absorbed and exhausted.^11^ The SEM images of the porous fibers/paraffin composites were shown in Fig. S6.† It is clear that the porous fibers form conductive networks and retain its original 3D architecture in paraffin. In conclusion, the proper filling content, the synergistic effect of dielectric loss, interface polarization loss, magnetic loss and porous structure make the Ni/C porous fibers have excellent microwave absorption performance.

## 4. Conclusions

In summary, Ni/C porous fibers were successfully prepared *via* a simple *in situ* template method by using low-cost jute fiber as carbon source and template. The as-prepared Ni/C porous fibers showed hollow internal cavity and porous structure, and the Ni nanoparticles were relatively dispersed on the surface and hollow porous structure of the Ni/C porous fibers. Due to the proper filling content, the synergistic effect of dielectric loss, interface polarization loss, magnetic loss and porous structure, the synthesized Ni/C porous fibers exhibited excellent microwave absorption performance. Under the matching thickness (1.5–3.5 mm), all of the values of the RL peaks were below −20.0 dB. This method has the advantages of low cost, abundant resources, simple technology, good repeatability, and suitable for large-scale preparation, which can not only provide a new way to design other types of excellent microwave absorbing materials, but also offer an effective way to synthesize biomass nanocomposites.

## Conflicts of interest

There are no conflicts to declare.

## Supplementary Material

RA-010-D0RA06817A-s001
