# Supplementary material for: Preparation of Ni/C porous fibers derived from jute fibers for high-performance microwave absorption
Source: RSC Adv. 2020 Oct 6;10(60):36644–53. doi: 10.1039/d0ra06817a (PMC9057028; doi:10.1039/d0ra06817a)
Supplement: RA-010-D0RA06817A-s001 [file RA-010-D0RA06817A-s001.pdf]

Electronic Supplementary Material (ESI) for RSC Advances.
This journal is © The Royal Society of Chemistry 2020

Supporting Information

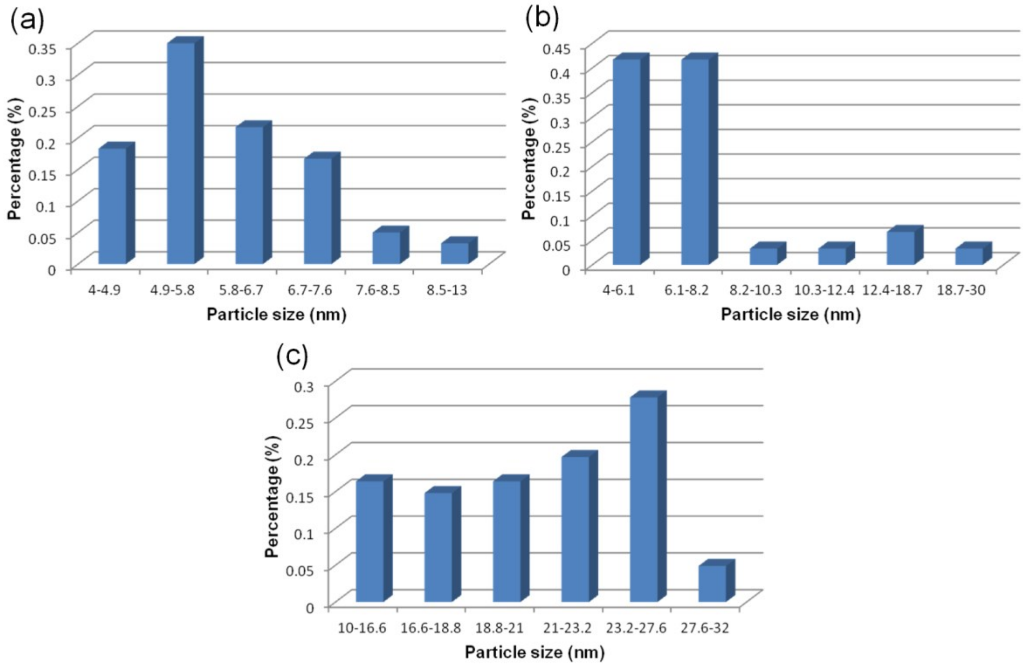

Fig. S1 The particle size distribution of Ni nanoparticles on (a) Ni/C-0.2, (b) Ni/C-0.5, and (c) Ni/C-1.0.

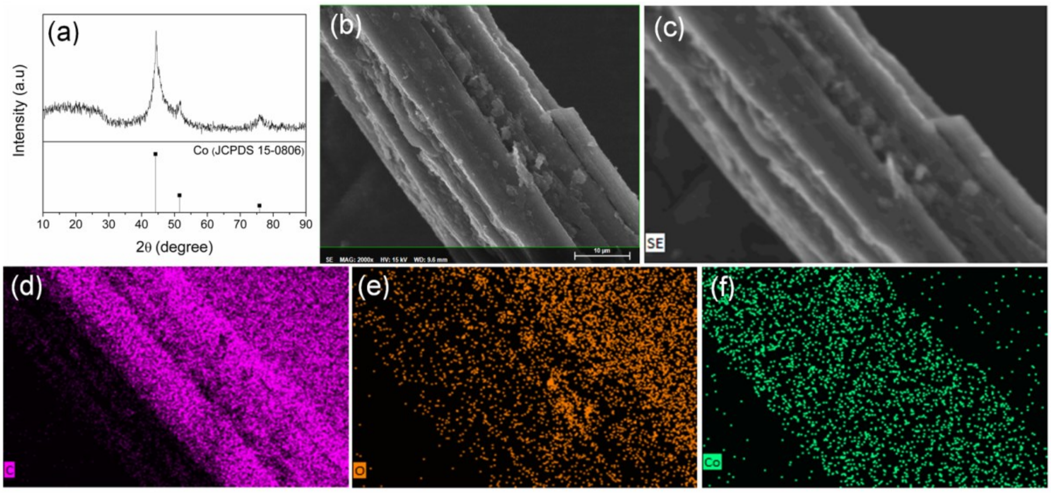

Fig. S2 (a) XRD pattern, (b) SEM image, and (c, d, e, and f) elemental mapping images of Co/C porous fibers.

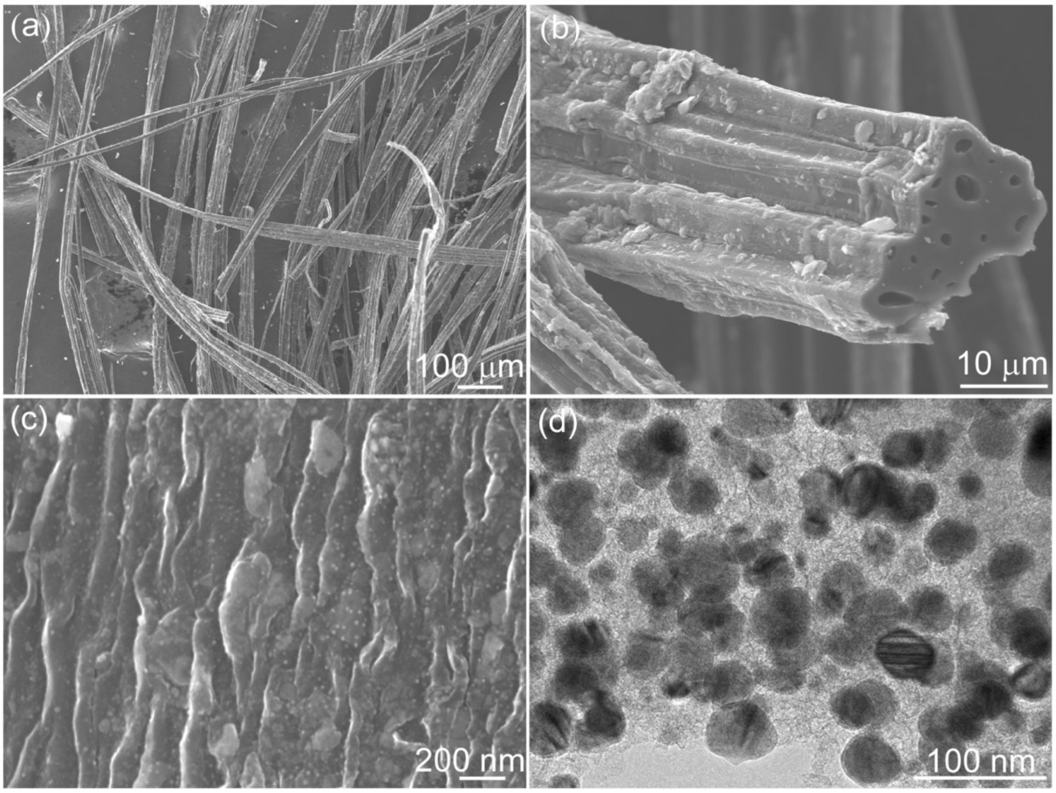

Fig. S3 SEM images (a, b, and c) and TEM image (d) of Co/C porous fibers in different magnification.

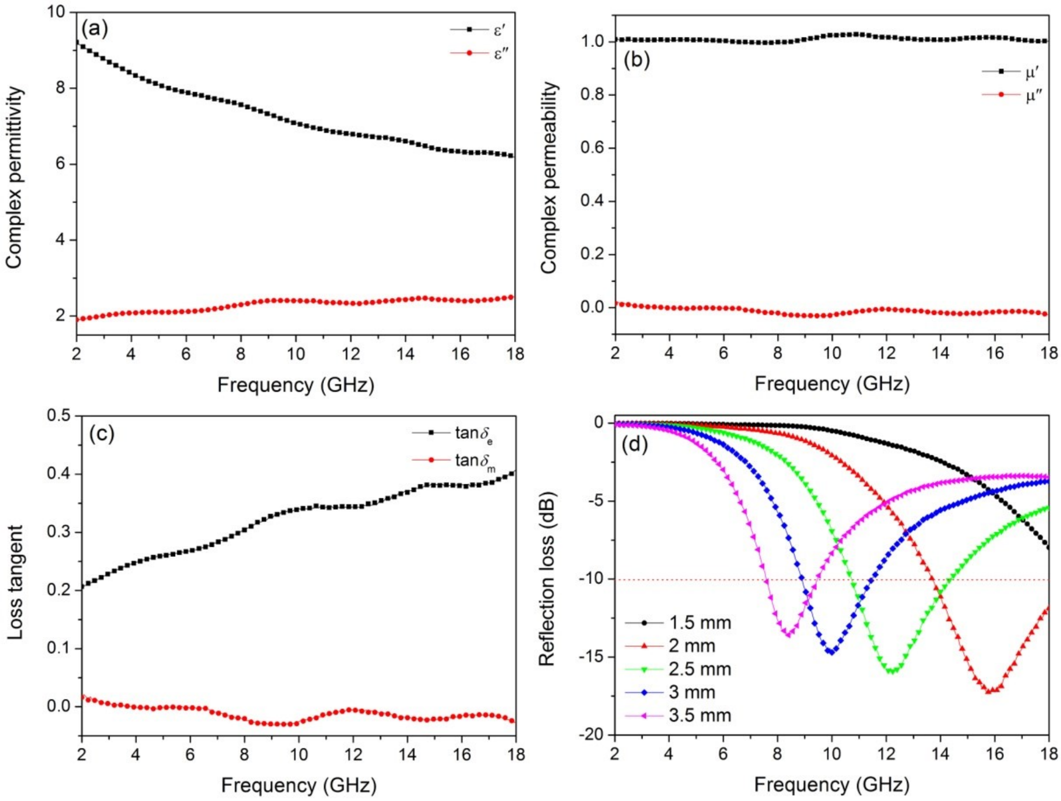

Fig. S4 Frequency dependence of (a) complex permittivity, (b) complex permeability, (c) loss tangent, and (d) reflection loss curve for the specimen with 25 wt% Ni/C-0.2 in paraffin.

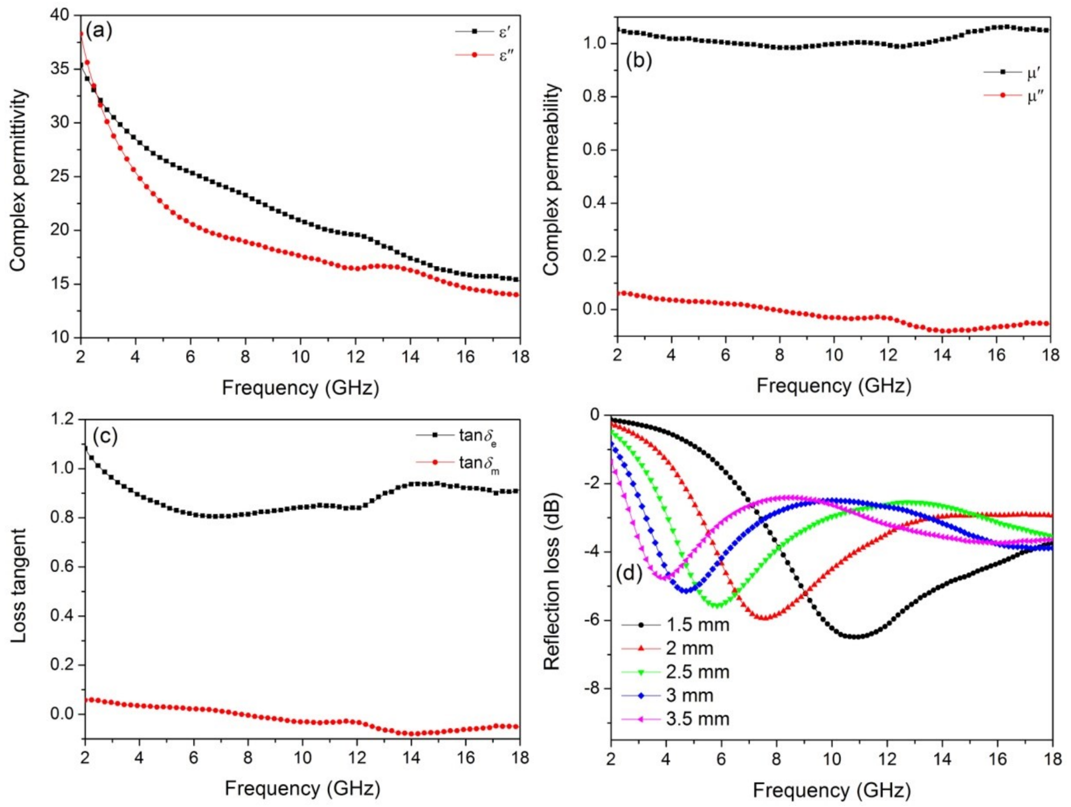

Fig. S5. Frequency dependence of (a) complex permittivity, (b) complex permeability, (c) loss tangent, and (d) reflection loss curve for the specimen with 50 wt% Ni/C-0.2 in paraffin.

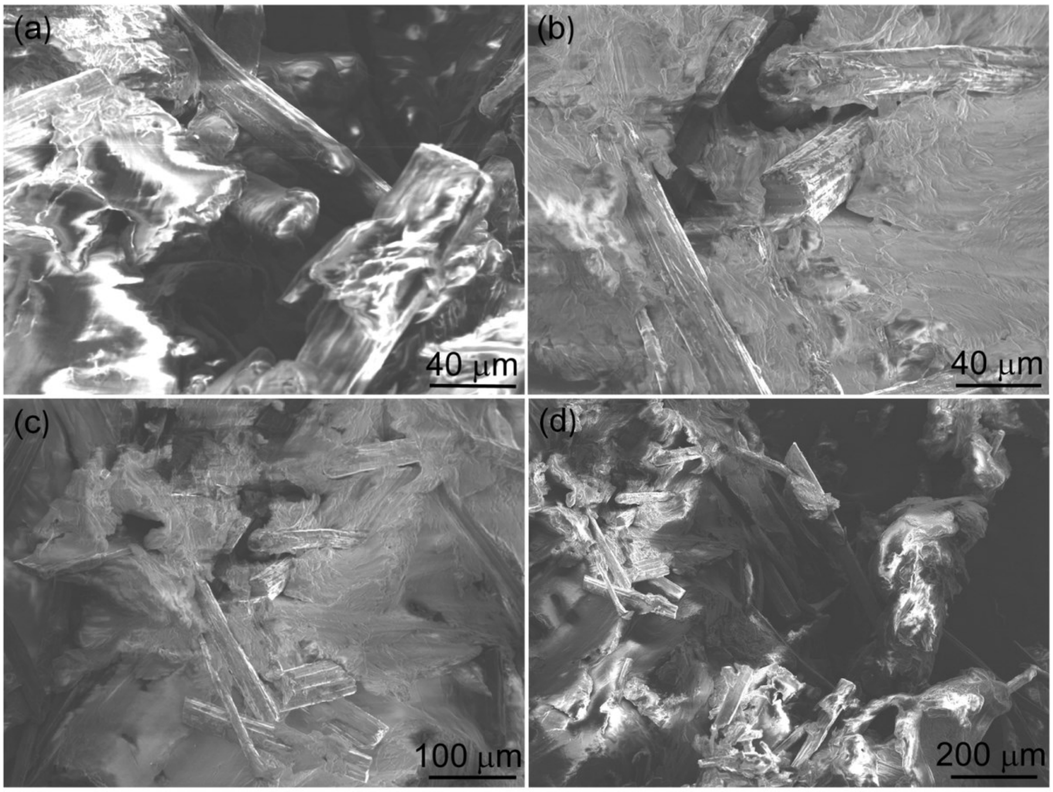

Fig. S6 SEM images of the porous fibers/paraffin composites in different magnification.
